# Age specific trends in mortality disparities by socio-economic deprivation in small geographical areas of England, 2002-2018: A retrospective registry study

**DOI:** 10.1016/j.lanepe.2021.100136

**Published:** 2021-06-07

**Authors:** Lucy Kraftman, Pia Hardelid, James Banks

**Affiliations:** a Institute for Fiscal Studies, London, UK; b Great Ormond Street Institute of Child Health, UCL, London, UK; c Department of Economics, University of Manchester, Manchester, UK

**Keywords:** Health inequality, Mortality inequality, Deprivation

## Abstract

**Background:**

Disparities in mortality rates according to socioeconomic position (SEP) have been rising in England. We describe the association between recent changes in socioeconomic inequality and trends in mortality disparities for different age and sex groups at small-area level in England.

**Methods:**

Vital registration data from the Office for National Statistics on resident population size and number of deaths in each Lower Super Output Area (LSOA) in England from 2002 to 2018 were stratified by sex and 5-year age group. We grouped LSOA into ventiles of the Index of Multiple Deprivation (IMD), our indicator of SEP. We examined time trends in smoothed mortality rates, using 3 year moving averages for the period 2003-2017, by age across the IMD distribution. We measured mortality inequalities using the ratio of mortality rates between different deprivation groups. We calculated mortality rate ratios between the most and the least deprived 10% of areas (Total Inequality) and between the median and least deprived (Lower Inequality) 10% of areas by year, gender and age group, to examine where in the distribution of deprivation trends in mortality inequality arose.

**Findings:**

Among <1 year olds, the inequality in mortality rates between the poorest 10% of LSOAs and the richest 10% of LSOAs fell between 2003 and 2017 by 22•7% for men and 22•8% for women. The largest inequalities were observed among 40 to 54 year olds. This inequality increased over the study period – from 3•2 times higher mortality rates for men in the most as opposed to the least deprived 10% of LSOAs in 2003 to 3•3 times in 2017. The rise was from 2•4 to 2•6 for women. Age groups ≥65 years, who experience the highest mortality risk, had low but rising inequality. Men and women aged 65 to 79 living in the most deprived LSOAs had a mortality rate 1•9 times higher than the least deprived in 2003 but this had increased to 2•2 times higher for women and 2•3 times higher for men by 2017. This was due to rising inequality in both halves of the distribution – between the top 10% of LSOA and the middle, and between the middle and the bottom 10% of LSOA.

**Interpretation:**

Overall mortality inequality rose in England but there were substantial differences in the trends for specific age and sex groups. Infant and child mortality inequality fell. At older ages, mortality inequality rose across cohorts, although in different ways, as each cohort's exposure to life-course to labour market inequality has differed. Policy goals of reducing mortality inequality will be best met by a focus on the risk factors that are specific to particular age and deprivation groups.

**Funding:**

Economic and Social Research Council, through the ESRC Centre for the Microeconomic Analysis of Public Policy at the IFS. We gratefully acknowledge the support of the Nuffield Foundation, grant reference WEL/43603. The project has been funded by the Nuffield Foundation, but the views expressed are those of the authors and not necessarily the Foundation. Visit www.nuffieldfoundation.org. Research at UCL Great Ormond Street Institute of Child Health is supported by the NIHR Great Ormond Street Hospital Biomedical Research Centre.

**Research in Context**

**Evidence before this study**

Following a similar search strategy to Bennet et al (2018), we searched PubMed from its inception up to 7^th^ May 2019 using the terms “life expectancy” AND “inequality” AND (“trend” OR “decomposition”) AND (“England” or “United Kingdom”) and the websites of Office for National Statistics (ONS) and Public Health England (PHE) to update with any new publications since their original search. In addition, we ran searches exchanging “life expectancy” with “mortality”. We identified only one further study of mortality trends in England or the UK by socio-economic position. Although we found a few papers examining age-specific mortality inequality, we found no papers that explored differences at different parts of the socioeconomic distribution in England.

**Added value of this study**

We demonstrate that changes in mortality inequalities since 2003 vary by age and gender. We show how these trends can be accounted for by the changing experiences of the top 10%, median and bottom 10% of the socioeconomic distribution. Our method provides a more detailed picture than using life expectancy at birth or at later ages.

Infant mortality inequality has fallen in England and this has been driven by large improvements amongst the most deprived 10% of areas compared to the least deprived 10% of areas. For those of working age (say age groups here), we did not identify one systematic pattern of mortality inequality over time. In the groups where mortality inequality has increased, this has been due to the groups with median deprivation levels improving more rapidly than the most deprived 10%, rather than the least deprived 10% improving more rapidly than the median. In contrast, whilst we mortality inequality has also increased for individuals ages ≥65 years, but this was due to the least deprived 10% of areas experiencing larger reductions in mortality rates compared to the rest of the population.

**Implications of all available evidence**

Among infants, 57% of deaths occur in the first week after birth, largely due to conditions related to prematurity or congenital anomalies [see ref in main text]. The reduction in infant mortality inequality has been coincident with improvements to antenatal, neonatal and obstetric care. Due to the universal nature of services delivered through the NHS, these have been available to everyone, thus leading to improved outcomes in the most deprived areas. Smoking during pregnancy, a key risk factor for infant morbidity and mortality has also decreased during the study period.

For those of working age, risk factors including life-course exposure to risky behaviours are higher in more deprived areas. Research has hypothesised that stagnant wages and changes in in-work environments, correlated with the increases in inequality across areas, is linked with an increase in suicide rates and other avoidable causes of death since 2002.

Mortality inequality has risen amongst pensioners, driven by larger reductions in deaths in the most advantaged, compared to the middle. Although labour market inequalities are no longer relevant for this group, inequalities still exist in social care and health care– highlighting the potential mechanisms that link mortality and poverty for this age group. In addition, growing in-work poverty and inequality in past wages will permeate into later life through savings and asset accumulation. This is highlighted through the growth of private pensions and pension-age employment. An 80 year old in 2003 had been exposed to less total-income inequality than an 80 year old in 2016, for example. Life course exposures to other risk factors, such as smoking, in earlier time periods are also important to consider.

Infants stand apart as their exposure to environmental risk factors has a limited time frame other than through the health and health behaviours of women before and during pregnancy. The reduction in mortality inequality for infants could suggest that investments in high quality universally accessible NHS care, when teamed with extensive public health interventions targeting the main life-course risk factors for different generations, might reduce mortality inequality for other age groups. This relies on the actions of a number of different departments in government and local authorities, often currently under tight spending constraints.

Alt-text: Unlabelled box

## 1 Introduction

Despite long-term rises in life expectancy and falling mortality [1] in England, mortality rates have not been falling equally across the socioeconomic distribution [2]. Indeed, life expectancy for women in the most deprived groups has fallen by 0•3 years between 2012 and 2018 [3]. At the same time, inequality in income and wealth rose from the 1980s until the 2008 recession. The share of total income amongst the top 10% increased rapidly from 26•5% to 32•1%[4]. In 1985, the top 1% had 15•5% of the net personal wealth. In 2012, this had reached 19•9%. Coupled with this, local authority services have been cut by 20% since 2010 [5], which lower income individuals rely on more heavily than those on a higher income. Health inequalities have become high on the public policy agenda, following the first Marmot review into health inequalities in 2010 [6]. In 2020, the lack of progress made by the government in reducing health inequalities was highlighted in the follow-up 10-year progress report [3].

There is growing fear that the UK is heading in the same direction as the US [9] where deaths involving drug-or alcohol, or suicide have been rising, particularly for those without university degrees. Lewer et al. (2020) found that 35•6% of premature deaths were attributable to socioeconomic inequality [7]. Between 1993 and 2017, deaths due to suicide, or involving drugs or alcohol, increased by 74•8%, from 14•7 per 100,000 population to 25•8 per 100,000 population among males aged 45-54 years in England [7]. Research in the US has linked these deaths to a process of cumulative disadvantage for less educated groups [8]. Recent evidence in England has found that the causes of death for which socioeconomic disparities are greatest include deaths due to opioid and psychoactive drug use [10].

Although higher mortality in poorer relative to wealthier groups in England is well documented [11], there is little evidence regarding how mortality inequality according to socioeconomic status has evolved over time according to age and gender (see ‘Research in context’).

Separating trends by age allows a focus on birth-cohort specific outcomes, which can then be related to life-course exposures for the cohort up to the point when death occurs. Since mortality at any given age is a function of accumulation of exposures and risks up to that point [12], this life course perspective provides a more constructive and specifically targeted evidence base for policymakers. Moreover, this accumulation of risks over the life course in different cohorts will likely have been different at different places in the socioeconomic distribution. There has been little evidence in England on where the inequality in mortality arises within the socioeconomic distribution, and whether this is similar across age and sex groups.

We used national statistics data on death by age group and sex to analyse trends in mortality inequality according to socioeconomic status over time in England. Although the data predates COVID-19, the paper provides important context in terms of trends and inequalities in pre-pandemic age-specific mortality rates.

## 2 Methods

### 2.1 Data sources

We used annual data from the Office for National Statistics (ONS) on mid-year population estimates and deaths registered from 2002 to 2018 inclusive in England by single years of age. We selected this study period since Lower Super Output Area (lower super output areas LSOA) level mortality has been published for these years. We split the deaths according to gender, age group (ages <1, 1-4, 5-14, 15-24, 25-39, 40-54, 55-64, 65-79 and 80 plus), and by LSOAs across England. LSOAs are areas used for reporting of small area statistics by the ONS, and have populations of 1,000-3,000 people. There are currently 32,844 LSOAs in England.

We used the Index of Multiple Deprivation (IMD) as a measure of socioeconomic deprivation, partly because LSOA level poverty or income data are not available, and partly because a broad deprivation index is important in capturing health and mortality risk factors. The IMD is the official measure of area-level deprivation in England and includes seven subdomains; employment, income, education, health, crime, housing and environment [12]. There have been multiple versions of the IMD during the study period; we applied the IMD closest to the year of interest in the analyses (2002 to 2005 (inclusive) used IMD 2004, 2005 to 2008 used IMD 2007, 2009 to 2012 used IMD 2010 and 2013 onwards used IMD 2015. We created an IMD that excluded the health subdomain (which captures measures that highly correlate with mortality) to avoid overstating the relationship between socioeconomic deprivation inequalities and mortality. To do this we constructed the weighted sum of the six remaining subdomains, using the same relative weights of those six subdomains as in the full IMD measure.

### 2.2 Statistical methods

We grouped deaths and resident population counts into ventiles of deprivation based on the ranked IMD scores of the LSOAs – each group included 5% of the LSOAs according to relative deprivation. This approach accounted for changes in LSOA deprivation ranks over time and avoided biases due to shrinking or growing number of LSOAs by always considering similar size groups. We calculated sex and age-group specific mortality rates by LSOA ventiles and year with 95% confidence intervals (CI). These CI's were calculated using the standard formula of  $\bar{x}\;\pm 1.96*se$, where $\bar{x}$ is the sex- and age-group specific mean mortality rate in each ventile-year and *se* is the square root of the standard deviation divided by the population size for the group.

We smoothed the annual rates using 3-year moving averages of mortality rates, hence creating an effective study period of 2003-2017. We age-standardised mortality rates using the most recent population data, reweighting the mortality rates so that the population of each 5-year age band represented the same proportion of the wider group (as defined in data sources) over time (e.g. 25-29 is a 5-year age band in the wider group of 25 to 39 year olds). This standardisation accounted for changes in the age structure within the broader group in each year in order to make this equal to their relative size in 2018. This is an important adjustment in an ageing population where, for example, the proportion of older individuals within the 40-54 age group has grown over the study period, thus raising the average age and the average mortality risk of the unstandardised group.

We first examined mortality inequalities for all ages, by sex, and then by age and sex. We plotted mortality rates by year, sex, age-group and ventiles of IMD to examine changes in mortality inequalities over time. All mortality rates were calculated per 1000 population. We plotted these rates in the first and last year of the study. We compare ventiles to give the most detailed picture feasible with our number of observations. We defined changes in mortality inequality as the percentage change in the differences in mortality rates over the study period. We also calculated mortality rate ratios between different IMD ventiles by year. We defined an index of Total Inequality (TI) as the mortality rate ratio comparing the most deprived 10% of LSOAs to the least deprived 10% of LSOAs. A ratio of one indicates no difference between the two groups, a ratio above one indicates higher mortality rates in the more deprived areas. We also defined an index of Lower Inequality (LI) as the mortality rate ratio comparing the most deprived 10% of LSOAs to the median 10% of LSOAs. Likewise, an index of Upper Inequality (UI) was defined as the mortality rate ratio comparing the least deprived 10% of LSOAs to the median 10% of LSOAs. Note that UI = TI / LI. We calculated TI ratios for each age group and the absolute number of excess deaths per year accounted for by TI in the first and last year of the study (2003 and 2017). This was calculated as the absolute difference in the age-adjusted mortality rate between the most and least deprived 10% of LSOAs, multiplied by the number of deaths in the most deprived 10% of LSOA and presented as number of deaths per 1,000 population. We also compared differences in changes in mortality inequality between males and females by comparing the percentage change in the mortality rate ratio comparing men and women in the most deprived compared to the least deprived LSOAs from 2003 to 2017.

To estimate confidence intervals on the mortality rate ratios and changes we fitted separate Poisson regression models for each gender and age group. We regressed LSOA level mortality rates on a full set of interactions of IMD decile indicators and year indicators. TI, LI and UI for each year were then calculated from the ratios of regression coefficients (e.g. TI_t_ = *β*_10t_/*β*_1t_ and UI_t_ = *β*_5t_/*β*_1t_) and t-tests and confidence intervals for each index were constructed. We also performed t-tests and constructed confidence intervals for the change of each ratio over the study period, and for the difference between UI and LI in each year of the study period.

### 2.3 Role of funding source

The sponsor of the study had no role in study design, data collection, data analysis, interpretation or writing of the report. All data are publicly available by the ONS and so all authors had full access. The corresponding author was responsible for submitting the Article for publication.

## 3 Results

This study included all deaths registered in England between 2002 and 2018 (*n*= 9.21 million), of which 51•8 % were in females, 48.2% were in males, and 0•1% (*n*=56,432) occurred among infants.

### 3.1 Summary of all-age disparities

#### 3.1.1 Changes in mortality inequality at all ages from 2003 to 2017

**Fig. 1:**
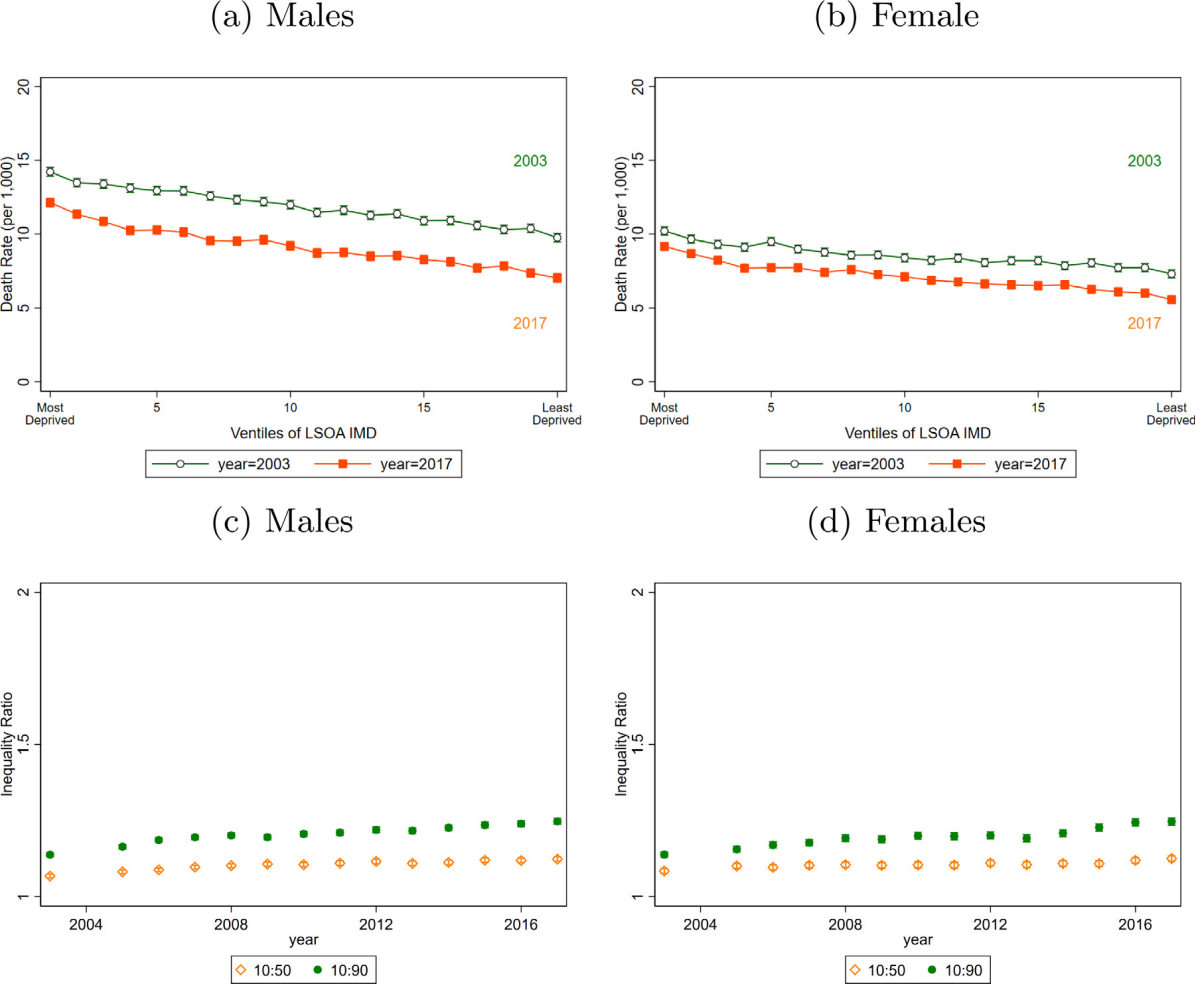
Death Rates and Death Rate Ratios for All-Ages, by decile or ventile of deprivation and sex from 2003 to 2017 Data from Office of National Statistics (ONS). Three-year moving average mortality rates are calculated. The mortality rates are age adjusted for all years using the most recent population data, 2018. In Panel a) and b) plot death rates across LSOA groups ranked by their deprivation level (according to the IMD). The vertical lines represent 95% confidence intervals from a Poisson model. In Panel c) and d) Death Rate Ratios are shown over time. The ratios are calculated by taking the average death rate for the LSOA that fall in the relevant decile and dividing (e.g. divide the average death rate for all LSOA in the bottom IMD decile by the average death rate for all LSOA in the top IMD decile to get the 10:90 ratio). The 50th decile represents the median 10% of LS. The 10:90 ratio is TI as defined in the methods section. The 10:50 ratio is the LI ratio.

Mortality rates fell for all IMD ventiles of LSOA between 2003 and 2017 but they decreased further in both relative and absolute terms for the least deprived areas (Fig. 1a, 1b). TI increased by 0.20 (CI (95%) = [0.12, 0.27]) for women, from 1.32 to 1.54 between 2003 and 2017. The LI ratio increased by 0.06 [0.00, 0.12] from 1.20 to 1.28. and UI increased by 0.10 [0.04, 0.17] from 1.11 to 1.21. For men, TI grew by 0.22 [0.15, 0.29] from 1.38 in 2003 to 1.63 in 2017. LI increased by 0.11 [0.06, 0.16] from 1.18 to 1.31 and UI rose by 0.07 [0.01, 0.13] from 1.16 in 2003 to 1.24 in 2017 (Table 1, Fig. 1c, 1d).

**Table 1:** TI in 2003 and 2017 and changes in TI, UI, LI in the study period.

	Age groups									
	0 to 1	1 to 4	5 to 14	15 to 24	25 to 39	40 to 54	55 to 64	65 to 79	80 plus	All ages
*Male*										
**Panel A: Total Inequality**										
TI in 2003	2.380	2.150	1.920	1.417	2.600	3.205	2.667	1.853	1.05	1.375
Deaths TI accounted for, 2003	6.107	0.212	0.08	0.284	1.133	4.024	10.588	22.637	27.431	3.782
TI in 2017	1.839	1.962	1.424	1.378	2.192	3.306	2.771	2.268	1.442	1.631
Deaths TI accounted for, 2017	3.181	0.128	0.034	0.158	0.788	3.548	8.179	22.554	47.666	4.544
**Panel B: Changes, 2003-2017**										
Change in TI (10 to 90)	**-0.541**	0.181	**-0.496**	-0.039	**-0.368**	0.101	**0.104**	**0.416**	**0.273**	**0.220**
	[-0.61,-0.48]	[-0.50,0.12]	[-0.84,-0.15]	[-0.16,0.08]	[-0.53,-0.20]	[-0.04,0.24]	[0.04,0.17]	[0.39,0.44]	[0.26,0.28]	[0.15,0.29]
Change in LI (10 to 50)	-0.03	**0.484**	0.021	0.056	**-0.260**	**-0.154**	-0.020	**0.149**	**0.155**	**0.109**
	[-0.09,0.17]	[0.18,1.78]	[-0.27,0.31]	[-0.04,0.16]	[-0.37,-0.15]	[-0.22,-0.08]	[-0.06,0.02]	[0.13,0.17]	[0.15,0.16]	[0.06,0.16]
Change in UI (50 to 90)	**-0.275**	-0.371	-0.361	-0.090	-0.011	**0.181**	**0.077**	**0.141**	**0.083**	**0.071**
	[-0.31,-0.24]	[-0.55,0.19]	[-0.62,0.10]	[-0.20,0.02]	[-0.11,0.09]	[0.10,0.26]	[0.04,0.12]	[0.12,0.16]	[0.08,0.09]	[0.01,0.13]
*Female*										
**Panel A: Total Inequality**										
TI in 2003	2.290	1.924	2.026	1.297	2.046	2.353	2.185	1.847	1.161	1.322
Deaths TI accounted for, 2003	4.599	0.16	0.072	0.087	0.425	1.89	5.104	14.638	18.916	2.423
TI in 2017	1.767	1.789	1.599	1.317	2.032	2.58	2.362	2.217	1.396	1.543
Deaths TI accounted for, 2017	2.391	0.097	0.036	0.054	0.367	1.865	4.650	14.861	35.348	3.141
**Panel B: Changes, 2003-2017**										
Change in TI (10 to 90)	**-0.523**	-0.135	**-0.427**	0.020	-0.015	**0.227**	**0.177**	**0.370**	**0.235**	**0.197**
	[-0.59,-0.46]	[-0.43,0.16]	[-0.85,-0.00]	[-0.16,0.21]	[-0.21,0.18]	[0.10,0.35]	[0.11,0.24]	[0.34,0.40]	[0.23,0.24]	[0.12,0.27]
Change in LI (10 to 50)	**-0.240**	-0.481	0.158	-0.097	**-0.347**	0.037	0.015	**0.176**	**0.092**	**0.060**
	[-0.29,-0.19]	[-0.75,0.22]	[-0.19,0.50]	[-0.23,0.04]	[-0.50,-0.20]	[-0.04,0.11]	[-0.03,0.06]	[0.15,0.20]	[0.08,0.10]	[0.00,0.12]
Change in UI (50 to 90)	**-0.137**	**0.226**	-0.408	0.143	**0.246**	**0.101**	**0.094**	**0.089**	**0.116**	**0.102**
	[-0.18,-0.09]	[0.03,0.42]	[-0.71,0.10]	[-0.04,0.33]	[0.11,0.38]	[0.04,0.14]	[0.66,0.11]	[0.06,0.11]	[0.11,0.12]	[0.04,0.17]

Note: Data from the Office of National Statistics (ONS). Three-year moving average mortality rates are calculated. The mortality rates for each broad age group are age adjusted sing the most recent population data, 2018, to keep the age composition constant over time. ‘Deaths TI accounted for’ shows the difference in the deaths (per 1,000) in the bottom and top 10% of LSOA in 2003 and 2017. TI, LI and UI are defined in the Methods section. TI, UI, LI, and changes in these over time are calculated from Poisson regression coefficients as described in the Methods section. Coefficients in bold are statistically different to zero at the 95% level. Intervals reported below the coefficients are 95% confidence intervals.

### 3.2 Differences by age group

#### 3.2.1 Infant mortality

**Fig. 2:**
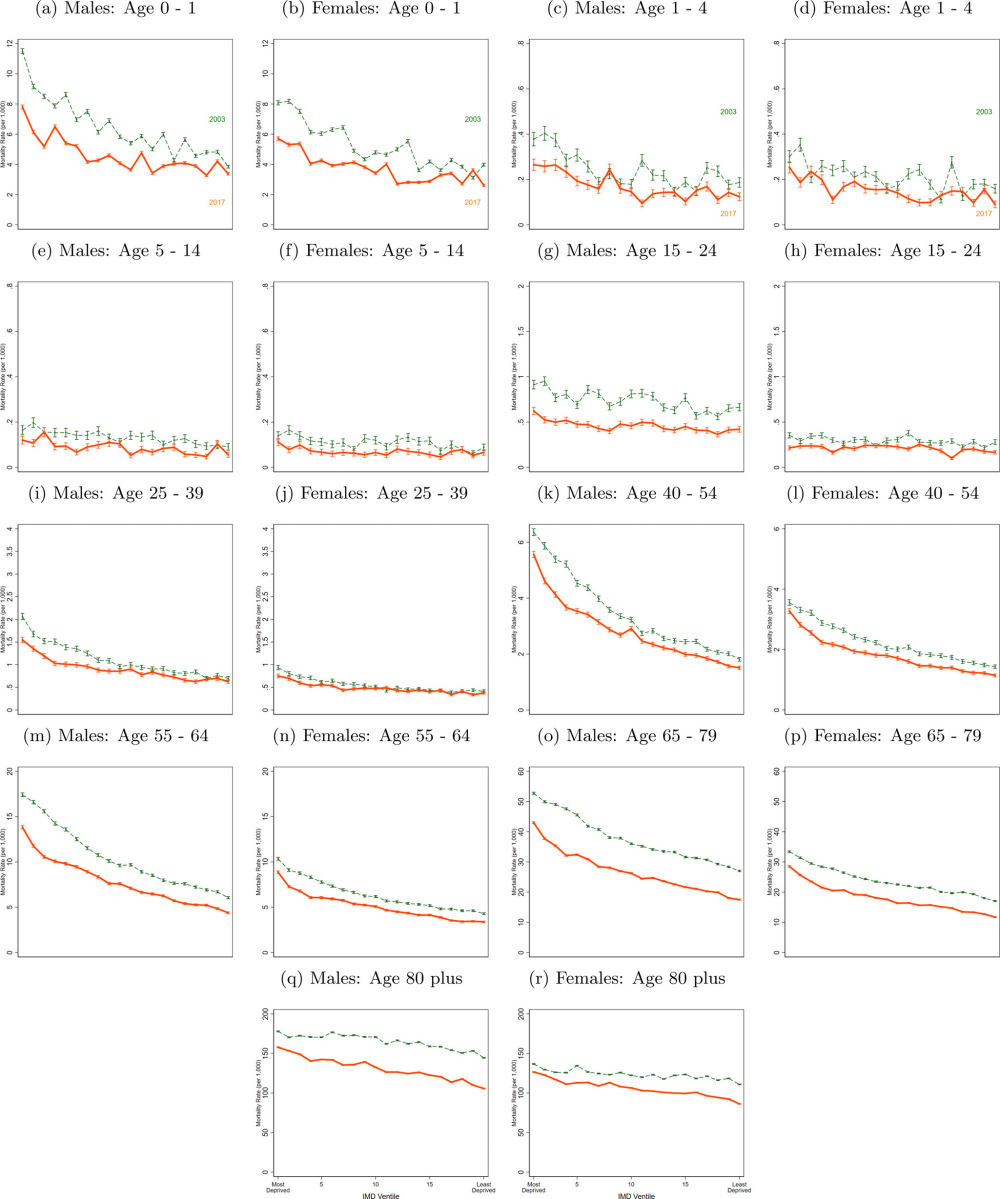
Death Rates (Deaths per 1,000 population) for selected Age Groups, by ventile of deprivation and sex in 2003 and 2017 Data from Office of National Statistics (ONS). Three-year moving average mortality rates are calculated. The mortality rates are age adjusted for all years using the most recent population data, 2018 to keep the age composition of each group constant over time. All panels plot death rates across LSOA groups ranked by their deprivation level (according to the IMD). The vertical lines represent 95% confidence intervals

**Fig. 3:**
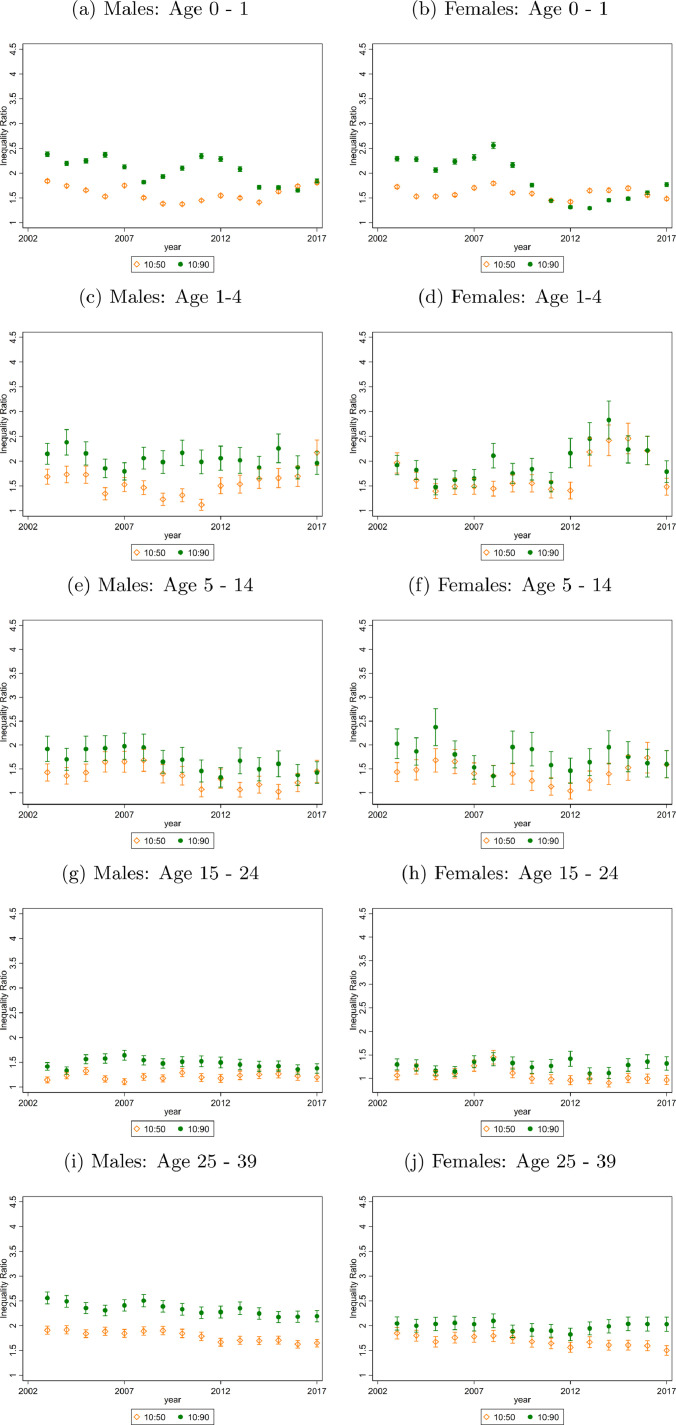

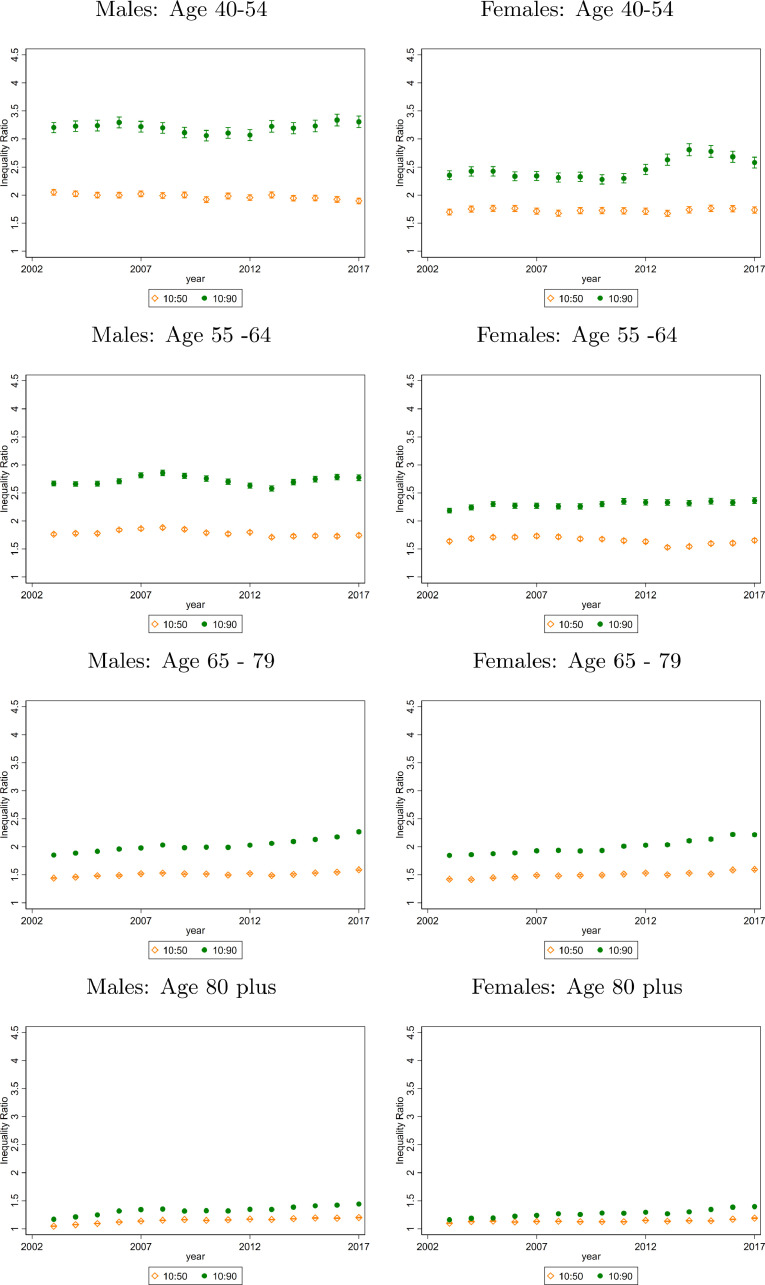
Death Rate Ratios for differing Age Groups, by decile of deprivation and sex from 2003 to 2017 Data from Office of National Statistics (ONS). Three-year moving average mortality rates are calculated. The mortality rates are age adjusted for all years using the most recent population data, 2018 to keep the age composition of each group constant over time. Death Rate Ratios are shown over time. The ratios are calculated by taking the average death rate for the LSOA that fall in the relevant decile and dividing (e.g. divide the average death rate for all LSOA in the bottom IMD decile by the average death rate for all LSOA in the top IMD decile to get the 10:90 ratio). The 50th decile represents the median 10% of LSOA. The 10% decile represents the top 10% of LSOA, i.e. the least deprived and the 90% decile represents the bottom 10% of LSOA, i.e. the most deprived. The 10:90 ratio is TI as defined in the methods section. The 10:50 ratio is the LI ratio.

For both sexes, absolute mortality inequality in infants reduced since 2003. (Fig. 2a and 2b). For boys, the infant mortality rate in the most deprived LSOAs fell from 11.51 [11.34, 11.67] to 7.80 [7.66, 7.93] between 2003 and 2017, compared to a fall from 3.85 [3.75, 3.94] per 1,000 population to 3.38 [3.29, 3.47] in the least deprived areas. Overall, there was a statistically significant decrease in TI of 0.54 [0.48, 0.61], from 2.38 to 1.84. For girls living in the most deprived 5% of LSOAs, the mortality rate was 8.08 [7.94, 8.21] in 2003 and fell to 5.71 [5.60, 5.83] by 2017. In the least deprived LSOAs in 2003 the mortality rate was 3.99 [3.89, 4.08] and in 2017 was 2.62 [2.54, 2.70]. TI fell by 0.52 [0.46, 0.59] from 2.29 to 1.76. (Table 1, Fig. 3).

#### 3.2.2 Children and young people

There were no clear patterns in mortality inequality trends at ages 1-4, 5-14 or 15 to 24 years, the age groups with the lowest mortality rates, although as with all other age groups mortality rates fell at all ventiles of deprivation. TI did fall significantly however for ages 5-14, by 0.50 [0.15, 0.84] for boys and 0.42 [0.00, 0.85] for girls.

#### 3.2.3 Working age adults

TI for men aged 25 - 39 years was 2.6 in 2003 and fell to 2.19 in 2017, a decrease of 0.37 [0.20, 0.53]. The change in LI was -0.26 [-0.37, -0.15] whereas UI did not change. For women aged 25 to 39, TI was at 2.05 in 2003 and at 2.03 in 2017, a fall of 0.02 [-0.21, 0.18]. Despite almost no change in TI, LI fell by 0.35 [-0.50,-0.20] but this was counterbalanced by an increase in UI of 0.25 [0.11, 0.38] (Table 1, Fig. 3).

Mortality disparities were largest among men and women aged 40 to 54 and 55 to 64. In 2003, a 40-54-year-old man in a most deprived 5% of LSOA was 3.52 times more likely to die than a 40-54-year-old man in a least deprived 5% LSOA. By 2017, this increased to 3.69 times more likely (Fig. 2). Within the most deprived areas, the inequality increased, as shown by the gradient between the dashed 2003 line and solid 2017 line for these ventiles in Fig. 2 – the poorest 5% LSOA have not kept up with improvements in the rest of the bottom half of the deprivation distribution. For this age group there was no statistically significant change in TI for males, LI fell by 0.15 [0.08, 0.22] and UI rose by 0.18 [0.10, 0.26]. For females, TI rose by 0.23 [0.10, 0.35], LI did not rise significantly and UI increased by 0.10 [0.04,0.14] for the period as a whole despite having fallen in the last four years (Fig. 2, Fig. 3, Table 1). Those aged 55 to 64 have lower TI than 40 to 54-year-olds but higher than older ages. The 2003 TI levels of 2.67 for men and 2.19 for women increased by 0.10 [0.04, 0.17] and 0.18 [0.11, 0.24] respectively by 2017.

#### 3.2.4 Older adults

Amongst 65 to 79-year olds, a man in the most deprived 5% of areas had a mortality rate of 52.73 [52.37, 53.08] in 2003 and 42.97 [42.65, 43.29] in 2017. TI in 2003 was 1.85 but had increased by 0.42 [0.39, 0.44] by 2017. This was a statistically significant change due to increases in both LI and UI. The mortality rate for women in the 5% of most deprived areas was 33.38 [33.10, 33.66] in 2003 compared to 28.51 [28.25, 28.77] in 2017. TI was 1.85 in 2003 and increased by 0.37 [0.34, 0.40] by 2017. Both LI and UI increased although the change in LI was much larger in magnitude. (Figs. 2, 3, Table 1). For women, excess deaths due to inequality increased over our sample period for all age groups over aged 65 (Table 1, panel A).

Among the over-80s, TI ratios were 1.44 and 1.40 for men and women respectively in 2017. High mortality rates in this age-group meant that for men, the TI corresponded to 47.66 more deaths per 1,000 population in the 10% of most deprived LSOA and the TI for women corresponded to 35.35 more deaths per 1,000 population (Table 1). The LI and UI were 1.10 and 1.15 and increased by 0.09 [0.08, 0.10] and 0.12 [0.11, 0.12] respectively over the study period. For males, the LI increased by 0.16 [0.15, 0.16] and UI by 0.08 [0.08, 0.09] (Figs. 2, 3, Table 1). For men, the 80 plus age group is the only group where excess deaths due to inequality increased over our sample period (Table 1, panel A).

## 4 Discussion

### 4.1 Summary of key results

Inequalities in mortality increased in England over the study period. For males, the total rate of deaths in poorest 10% of LSOAs was 1.4 times the rate in the richest 10% LSOA in 2003. By 2017, this had increased to 1.6 times. For females, the rate of deaths in the poorest 10% of LSOA in 2003 was 1.3 times the rate in the richest 10% of LSOA, rising to 1.5 times by 2017.

We found diverging trends in mortality inequality according to age in England since 2003. For <1-year olds, total inequality fell during the study period whereas it increased for age groups aged 40 years and above. 40 to 64-year olds had low mortality rates compared to the older ages but the highest levels of mortality inequality, which were increasing. These trends in total inequality concealed important differences in where the inequality was occurring. Whilst in the age groups 65 and over the increase in inequality arose both in the top half and the bottom half of the socioeconomic distribution, in age groups under 65 there were often different trends in inequality in each half of the distribution, and also important differences within the bottom 10–15% of LSOA for older working age adults.

### 4.2 Strengths of study

Aggregate national mortality data, which are freely available online, cover the whole of England, minimise selection bias due to their universal coverage, and can be analysed without the time required to seek approval from data providers required for individual-level datasets. We used age-specific mortality rates rather than life expectancy as an indicator to compare mortality between population subgroups. As Baker, Currie & Schwandt [13] discuss, life expectancy most commonly refers to life expectancy at birth, but when mortality rates at different ages change differentially, trends in life expectancy at given ages can also differ. Life expectancy at birth is highly sensitive to infant mortality, such that changes in infant mortality can sometimes obscure the picture of changing distribution of deaths at later ages – if infant mortality rates are improving and so are mortality rates at old age but mortality rates are rising in midlife, the single Fig. given at life expectancy at birth may not reflect this [13].

By examining mortality inequality in different age groups, which have been exposed to different risk factors across their life course, and considering the full distribution of IMD, we were able to explore different reasons for the mortality inequality that we observed. Presenting cross-sectional mortality rates in different years for different age groups allows life course mechanisms to be considered. When discussing drivers of different inequality at different ages, a combination of longitudinal life course explanations and current policy effects will matter. Whilst our study cannot infer causal links, breaking mortality rates down into age groups and observing differing trends for different cohorts across the full IMD distribution allows an indication of potential pathways to explore, or to rule out. For example, when focussing on increasing mortality inequality at age 45 or below, changes in pension policy are unlikely to be a key part of the picture. This would not be possible through an analysis of overall life expectancy.

Our study examined changes in mortality inequality at different positions within the IMD distribution. This analysis made it possible to break down whether an increase in mortality inequality is due to the most deprived groups experiencing less rapid decreases in mortality (or indeed stagnant or rising mortality) compared to the rest of the population, or the majority of the population failing to keep up with the least deprived groups. The distinction between the two different mechanisms underlying the same overall increase in inequality is important when considering potential causes of inequality and thus policy implications.

### 4.3 Weaknesses of study

We used IMD as an indicator of socioeconomic status in this study. While IMD is a commonly used indicator of deprivation in health research in England, IMD measures are not wholly comparable over time as some elements change, such as inclusion of new benefits. Moreover, even the most recent IMD measures rely heavily on the 2011 Census (see ONS, 2015 for details of the measure) and therefore may not capture up-to-date deprivation levels in a particular LSOA. Our analysis also uses IMD rank as a relative measure of LSOA compared to others. Hence an LSOA could move up or down the rank ordering if other LSOAs change in deprivation, without any changes in that one LSOA itself. In addition, it may be that being the least deprived is not exactly equivalent to being the most affluent, for example, having low numbers of individuals claiming Job Seekers Allowance may mean the area is not deprived but does not necessarily mean the area is amongst the wealthiest in society; this was something we were unable to capture.

Moreover, the ecological fallacy may apply to our study, where living in a deprived area according to IMD, does not mean every individual themselves are deprived. Assessing this would require individual level data from tax records (to measure income) or detailed individual level data from the Census on education level for example, linked to mortality records. We highlight that IMD is therefore capturing local level deprivation rather than individual. However, as LSOAs are so small, the area measures do correlate more highly with individual deprivation than larger areas, such as Local Authorities where heterogeneity within each area is large [14]. We also did not focus on causes of death due to the lack of openly available data at LSOA level. However, especially at older ages, underlying cause of death may not be suitable since many older people have multiple long-term conditions that may contribute to death. Additionally, for deprived groups, whilst the cause of death may be informative, there may have been an accumulation of different risk factors leading to those higher mortality rates [15].

Whilst our analysis has used summary statistics breaking down inequality into the components coming from the top and bottom halves of the distribution there may be other finer patterns in the shape of inequalities over time within age groups that our analyses did not capture. Furthermore, our measure of total inequality across all age groups is not fully captured by the sum of our total inequality measures across age groups due to between age-group inequalities. Finally, we examined mortality data according to date of registration, rather than date of occurrence. Since a death in England cannot be registered without a known cause of death, this can cause substantial delays (in some cases >12 months). These delays particularly affect deaths among young adults, and means that for age groups between 10 and 45, a substantial proportion of deaths registered in a particular year have occurred in the previous year or years [16].

### 4.4 Interpretation

In infants, 72% of deaths occur in neonatal period (first month of life), and 97% of these deaths occur in hospital [1]. The observed infant mortality reduction during the study period was most likely due to recent improvements in obstetric and neonatal care (e.g. steroid injections for women at risk of premature birth, surfactant replacement for premature babies to prevent respiratory distress) [17,18]. These interventions are delivered through the NHS and therefore benefit all babies, independent of their socioeconomic status, since >99% of births in England are under NHS care. NHS care is free at the point of need, therefore if most of the care for an age group is provided through very intensive NHS care, mortality inequality for this age group would be expected to be lower. Further, the prevalence of smoking during pregnancy has decreased in England during the study period [19]. Smoking and exposure to tobacco smoke is a key risk factor for premature birth, intrauterine growth restriction, and some congenital anomalies, which in turn increase the risk of infant mortality [20], [21], [22]. This decrease in infant mortality has occurred despite limited reduction in child poverty: the percent of children in low-income households has remained constant at around 30% since 2000 [23]. Therefore, the likely cause of this reduction in infant mortality inequality is NHS improvements and reduction in smoking rates.

At older ages, life-course factors such as living environments, accumulation of wealth and labour market exposure, as well as health behaviours, are key risk factors for mortality [24]. By breaking down trends according to age group and according to LI and UI, we can see that single explanations for the underlying causes of increasing inequality are unlikely to be sufficient in explaining overall inequality. In working age adults, for example, when mortality inequality is largest, suicide, drug and alcohol overdoses and alcohol-related liver disease have been rising rapidly in the UK [25] and elsewhere. Suicide and poisonings are now the leading causes of death for men aged 35-49 and suicide deaths are at their highest since 2002 [26]. In the USA, Case & Deaton (2017) suggest that a process of cumulative disadvantage for the less educated [8] could be the cause of the increase in the US. Wage stagnation and increases in in-work poverty for those at the bottom of the income distribution [27], [28] in England could be relevant and our results show a steepening in the mortality gradient for those aged 40-54 and 55-64 between the bottom 5% of LSOAs and the bottom 10% LSOAs that would be consistent with this. The increase in UI for these age groups, however, suggests that other factors are also likely to contribute to increases in total inequality at these ages. Changes in the labour market across the whole socioeconomic status distribution, such as widening inequality in real wages for both men and women, as well as changes in life-course risk behaviours for mortality from other causes of death, should also be explored in future studies as potential explanations for increasing mortality inequality in this group.

While female mortality inequality between the ages of 40 and 64 was lower than for men in 2003, it rose more rapidly in this age group over the study period. This was a period in which inequalities in breast and cervical cancer screening which are relevant for the lead causes of death for this age group, whilst still present, have been falling [29]. Successive cohorts of women are now exposed to in-work risk factors for longer as a result of an increase in female labour force participation, which rose from 55% in 1971 to 71.4% in 2018, and a rise in the State Pension Age since 2010. Labour market related health risks may have therefore become increasingly relevant to woman, hence driving the increased inequality relative to men [28]. Further research is required into if and how increased labour market participation has causally affected mortality inequality for women.

There have been reductions in mortality rates but also growing mortality inequality in among people aged 80 years and above. At these ages, even small changes in inequalities can translate into large differences in the number of deaths between more vs less deprived areas since the excess death due to mortality inequality is the highest. Indeed, men over age 80 and women over aged-65 are the only groups where excess death due to inequality has increased over our sample period. On average, pensioner wealth has increased - the median net equivalized household income for pensioners had steadily grown from 85 to 108 (standardised at 100 in 2007/08) - within the study period [28]. Along with improving health technology (such as widespread use of statins and use of stents to reduce mortality from heart attacks [30]), and reductions in smoking prevalence across cohorts [31], this may help explain reductions in overall mortality rates. However, whilst there is less economic inequality between pensioners and those of working age, there has been increasing economic inequality amongst pensioners [28], reflecting rises in lifetime labour market inequality and changes in the welfare state. Despite benefit income growing relatively equally amongst pensioner income quintiles between 1980 and 2012, it remained almost the entire income for the bottom quintile, whilst the top had also seen very large growth in private pensions, savings and investments and earnings and self-employment income [28].

This widening of the economic distribution of economic resources at older ages at both the top and the bottom, reflecting life-time labour market, risk factors and wealth accumulation, is consistent with the large and significant increases in both UI and LI that we see in the mortality data for these age groups. Pension income inequalities have also become increasingly important for recent cohorts since life expectancy at age 65 has grown rapidly since the 1940s for women and 1970s for men. Individuals are therefore living on pension incomes for a considerably larger fraction of their lives than previously.

### 4.5 Policy Implications

Since trends in mortality inequality vary according to age group, gender and deprivation level, one important implication is that one single policy is unlikely to deliver reductions in overall mortality inequality. Instead, the different patterns across cohorts and deprivation groups point to a need to address health behaviours or other risk factors that different age-gender and socioeconomic groups are exposed to over their life-course. Multiple policies and policy targets are needed to address inequalities in mortality outcomes for different cohorts. In addition, policies that are not implemented directly for health or public health reasons might have unintended consequences for mortality inequalities across cohorts. The changes of the State Pension Age for women born between 1950 and 1960 is an obvious example, but the ‘triple lock’ rules for the protection of pension benefits will have differentially affected individuals depending on their age when introduced in 2010. This suggests the importance of a Health in All Policies approach [32,33] that considers health outcomes at different ages and levels of deprivation.

Life-course decisions and environments will have impacts on health inequalities throughout one's life since exposure to environmental hazards and health behaviours such as air pollution, poor diet, tobacco smoke and lack of exercise contribute to disease and mortality and are positively correlated with disadvantage [34]. Hence, population level health behaviour interventions such as those addressing the pricing or advertising of tobacco, alcohol or unhealthy food or those targeting physical health by reforming transport policy or promoting physical exercise, can potentially affect effects on mortality inequalities as long as they are targeted towards more deprived groups [35], [36], [37].

Since 2012 the statutory responsibility for many population level interventions and health improvements has fallen on local authorities. Thus, differential spending on public health by local areas may also be an important mechanism driving mortality inequalities. Most of the funding for public health initiatives has come from a ring-fenced public health grant. This grant has been decreasing year on year and continues to; from £3.47bn in 2016/17 to £2.07bn in 2020. Local authority spending on public health functions in the last two years has therefore reduced and is projected to reduce further from £3•47 billion in 2016/17 to £2•07 billion in 2020/21 [5]. Despite higher reliance on public spending amongst more deprived areas, local government spending on services saw higher cuts in more deprived areas than more affluent ones [5]. If public health spending influences mortality, cuts to local public health budgets may therefore increase the mortality inequality. Currie et al. (2018) shows that an increase in funding to the most deprived areas was associated with reductions in amenable mortality among men [38]. A Health in all Policies approach is needed to take seriously the potential impact that such changes in spending could have on health outcomes and to provide evidence on the trade-offs involved.

Although improvements in health and social care are needed to reduce mortality inequalities for those currently at older ages, addressing later life mortality inequalities needs to start early in life, and therefore take years to take effect. It is more efficient to target risk factors and equal preventative care measures for all than wait until the health of individuals deteriorate and then treat them [39]. Average hospital spending for an 89-year-old man is around three times higher than the average spending for a 70-year old and almost 9 times higher than a 50-year old [40]. Average spending per person is higher in more deprived areas, by 26% for 25-64 year olds, and by 35%for over 65s [40]. However, this does not control for differences in underlying medical needs. If underlying health needs are taken into account the relationship may well reverse – meaning that someone with the same need in a more deprived areas are getting less spending than someone who is less deprived [41], [42], [43] and this may well have changed differentially by age and with time. Trends in the older population groups predated, but will have been exacerbated by, the high COVID-19 mortality rates in the elderly population in the poorest areas [44].

## Conclusions

Mortality inequality above the age of 40 is increasing. In late middle age this is due to the most extremely deprived areas falling behind the rest of the population and the least disadvantaged areas accelerating away from the middle. At older ages it is due to a more general widening of the distribution, both in terms of the gaps between the bottom and the middle, and those between top and the middle. Despite these trends at older ages, and despite rising socioeconomic inequality more generally, inequality in infant mortality has been falling. A nuanced understanding of these trends, and their most likely causes, in addition to breaking down where inequality lies in the socioeconomic distribution crude indicators of overall inequality in life expectancy, is essential for policymakers looking to reduce health inequalities in the future. The methods in this study could also be usefully applied to understanding mortality inequalities in the face of aggregate health shocks such as the COVID-19 pandemic, which have had differential effects across the age and socioeconomic status distribution.

## Contributors

JB and LK worked on the study design, JB, PH and LK worked on the literature search, data interpretation and writing. LK also worked on the figures and data analysis.

## Declaration of Competing Interests

No authors have competing interests.
